# Associations between urinary heavy metal concentrations and blood pressure in residents of Asian countries

**DOI:** 10.1186/s12199-021-01027-y

**Published:** 2021-10-08

**Authors:** Yuki Mizuno, Hana Shimizu-Furusawa, Shoko Konishi, Tsukasa Inaoka, Sk Akhtar Ahmad, Makiko Sekiyama, Oekan S. Abdoellah, Budhi Gunawan, Rajendra Prasad Parajuli, Yukio Ikemoto, Tran Dinh Lam, Chiho Watanabe, Masahiro Umezaki

**Affiliations:** 1 Department of Human Ecology, Graduate School of Medicine, The University of Tokyo, 7-3-1 Hongo, Bunkyo-ku, Tokyo, 113-0033 Japan; 2 Department of Analytic Human Pathology, Nippon Medical School, 1-1-5, Sendagi, Bunkyo-ku, Tokyo, 113-8602 Japan; 3 Department of Human Ecology, Faculty of Agriculture, Saga University, 1 Honjo Machi, Saga, 840-0052 Japan; 4 Department of Occupational and Environmental Health, Bangladesh University of Health Sciences (BUHS), Darus Salam Mirpur-1, Dhaka, 1216 Bangladesh; 5 Health and Environmental Risk Division, National Institute for Environmental Studies, Onogawa 16-2, Tsukuba, Ibaraki, 305-8506 Japan; 6 Department of Anthropology, Faculty of Social and Political Science, Universitas Padjadjaran, Jl. Raya Bandung-Sumedang Km. 21 Jatinangor, Sumedang, West Java Province 45363 Indonesia; 7 Central Department of Zoology, Central Campus, Institute of Science & Technology (IOST), Tribhuvan University, Kritipur-1, Kathmandu, Nepal; 8 Institute for Advanced Studies on Asia, The University of Tokyo, 7-3-1 Hongo, Bunkyo-ku, Tokyo, 113-0033 Japan; 9 Center for Vietnamese and Southeast Asian Studies, Vietnam National University of Social Sciences and Humanities, 10-12 Dinh Tien Hoang, Ben Nghe Ward, District 1, Ho Chi Minh City, Vietnam; 10 School of Tropical Medicine and Global Health, Nagasaki University (NCGM Satellite), 1-21-1, Toyama, Shinjuku-ku, Tokyo, 162-8655 Japan

**Keywords:** Arsenic, Cadmium, Lead, Selenium, Blood pressure

## Abstract

**Background:**

Previous studies have suggested that exposures to heavy metals (arsenic, cadmium, lead, and selenium) may be associated with differences in blood pressure. However, the findings of these studies have been inconsistent. This study was performed to examine the associations between urinary heavy metal concentrations and blood pressure among residents of four Asian countries (Bangladesh, Indonesia, Nepal, and Vietnam).

**Methods:**

This cross-sectional study examined 1899 adults in four Asian countries. Urinary concentrations of heavy metals were measured by inductively coupled plasma mass spectrometry. A questionnaire survey was administered regarding individual characteristics. Anthropometric measurements (height and weight) were performed. Systolic and diastolic blood pressures were measured after a short rest. Multiple linear regression models were applied to investigate associations between urinary heavy metal concentrations and blood pressure after adjustments for age, sex, and body mass index.

**Results:**

The geometric means of the urinary concentrations of arsenic, cadmium, lead, and selenium were 84.6, 0.885, 2.09, and 16.5 μg/g creatinine, respectively. The urinary arsenic concentrations were slightly higher than those typically reported in non-polluted populations, while urinary cadmium, lead, and selenium concentrations were equivalent or slightly lower. The urinary lead concentrations were positively associated with both systolic and diastolic blood pressure, but urinary selenium concentrations were negatively associated with them.

**Conclusions:**

Variations in the urinary concentrations of lead and selenium were associated with blood pressure at low levels of exposure/intake.

**Supplementary Information:**

The online version contains supplementary material available at 10.1186/s12199-021-01027-y.

## Background

Blood pressure is defined as the pressure of circulating blood within arteries. Excessive energy or salt intake, lack of physical activity, and presence of psychological stress are major risk factors for developing high blood pressure [1–4]. Hypertension, the state in which blood pressure exceeds a certain threshold, is an important risk factor for various cardiovascular diseases. More than 1 billion people have been diagnosed with hypertension worldwide [5].

Exposure to toxic heavy metals, e.g., arsenic (As), cadmium (Cd), and lead (Pb), may be associated with individual variations in blood pressure [6, 7]. The biological mechanisms that drive heavy metal-induced blood pressure changes include Cd-induced nephrotoxicity [8–10], as well as increased cardiac output and vasoconstriction triggered by exposure of the sympathetic nervous system to Pb [11]. Furthermore, As, Cd, and Pb can damage endothelial cells by promoting inflammation and/or oxidative stress [12].

Selenium (Se), an essential trace element, may also affect blood pressure [13]. In humans, Se is a cofactor of glutathione peroxidase, a selenoprotein that acts as an antioxidative enzyme [14, 15], which can mitigate the toxicity of heavy metals by its antioxidative effects [16]. Glutathione peroxidase activity reduces lipid peroxidation, atherosclerotic plaque formation, and platelet aggregation [17–19]. Therefore, Se intake can reduce the risk of high blood pressure.

The results of several epidemiological studies investigating the relationships between blood pressure and exposure to As, Cd, Pb, and Se have demonstrated contradictory results. For example, several reports described positive associations between blood pressure and exposure to Cd or Pb even in regions with known sources of contamination [20–24]. However, other studies found no associations [20, 25] or negative associations [23, 26, 27]. While a positive association between As exposure and blood pressure in As-contaminated regions has been established [28–30], it remains unclear whether this association exists in non-contaminated settings. Furthermore, studies examining associations between Se status and blood pressure have reported negative associations [31, 32], positive associations [33, 34], and no associations [35–37]. These inconsistencies have not yet been fully explained. However, variables that vary among study populations (e.g., nutritional status) might confound the associations of toxic heavy metal exposure and Se intake with blood pressure.

Despite an investigation of the association between heavy metal exposure and blood pressure, the studies in non-contaminated settings are still scarce. Studies are also lacking in rural areas in Asia, where people would be more vulnerable to the heavy metal exposure due to their relatively poor nutritional status. Therefore, the aim of this study was to examine the associations between urinary concentrations of heavy metals (As, Cd, Pb, and Se) and blood pressure among residents of four Asian countries. Additionally, this study investigated differences in the associations among the four countries, which were thought to have different heavy metal exposure levels.

## Methods

### Study population and sampling

This cross-sectional study was conducted by examining 2241 adults in Bangladesh, Indonesia, Nepal, and Vietnam. Several farming communities with varied characteristics (e.g., reliance on subsistence farming and reliance on large-scale cash cropping) were selected in each country, and all de facto residents of each community were invited to participate in the study (see Table 1 for the characteristics of the target communities). We recruited individuals aged 18 years or older and had neither a high fever nor diarrhea on the survey days. Among the 2241 participants, blood pressure was not measured in 310, such that the final sample size was 1931.

**Table 1:** Characteristics of the target communities in this study

Country	Community	*n* (male, female)	Location	Main occupation
Bangladesh (*n* = 549)	BV	107 (51, 56)	Urban	Tertiary industry
	BJ	71 (39, 32)	Peri-urban	Farming (rice, vegetables, beans, and jute)
	BZ	60 (30, 30)	Peri-urban	Farming (rice, vegetables, and mango)
	BS	95 (42, 53)	Rural	Farming (rice, vegetables, and jute)
	BT	107 (57, 50)	Rural	Farming (rice, vegetables, and jute) and migrant labor
	BC	109 (42, 67)	Rural	Wage labor
Indonesia (*n* = 186)	P	100 (50, 50)	Rural	Farming (rice)
	S	86 (46, 40)	Urban	Various occupations (e.g., farming, construction industry, etc.)
Nepal (*n* = 700)	NC	169 (65, 104)	Rural	Farming (rice and vegetables) and commerce, wage labor, and migrant labor
	NP	123 (54, 69)	Rural	Migrant labor and farming (rice, mustard, and dairy)
	NW	161 (70, 91)	Urban	Commerce and wage labor
	NI	128 (59, 69)	Rural	Cash-crop farming (tea, cardamon, and broom grass)
	NK	119 (55, 64)	Peri-urban	Migrant labor, business, and farming (rice and mustard)
Vietnam (*n* = 496)	VC	134 (66, 68)	Rural	Cash-crop farming (coffee)
	VP	136 (53, 83)	Rural	Farming (rice and corn)
	VA	114 (58, 56)	Urban	Retail business and recycling industry
	VT	112 (53, 59)	Rural	Rice, shrimp, and dairy farming

*n*, number of participants

Using face-to-face questionnaire surveys, the participants were asked about individual characteristics (e.g., sex and age). Anthropometric measurements (height and weight) were also performed. Systolic blood pressure (*SBP*) and diastolic blood pressure (*DBP*) were measured by a digital sphygmomanometer or a mercury manometer after a short period of rest. Additionally, a spot urine sample was collected for each participant.

We asked the participants to collect their spot urine samples using a urine cup and dispensed the samples into screw-capped polypropylene tubes. The tubes were washed with 15% nitric acid and rinsed with ultrapure water prior to sampling. The collected urine samples were frozen with dry ice, then transported to Japan, and stored at −80°C until analysis.

Written informed consent was obtained from each participant before the survey. Ethics approval was obtained from the Ethics Committee of the Graduate School of Medicine at the University of Tokyo (No. 1505-(1)).

### Measurement of urinary heavy metal concentrations

Urinary concentrations of As (mass-to-charge ratio (*m/z*) = 75), Cd (*m/z* = 111), Pb (*m/z* = 208), and Se (*m/z* = 78) were measured by inductively coupled plasma mass spectrometry (Agilent 7500ce). In brief, each urine sample was diluted 20-fold with 1% nitric acid (60%; Wako, Osaka, Japan) and 2% 1-butanol (99.5%; Nacalai Tesque, Kyoto, Japan), then filtered through a 0.45-μm pore membrane. Urinary creatinine concentrations were measured using a spectrophotometric method based on Jaffe’s reaction. To adjust for the dilution effect, the urinary concentration of each metal was divided by the creatinine concentration.

Urinary Cd concentrations reflect long-term Cd exposure (i.e., more than 10 years) [38, 39], while urinary As concentrations reflect relatively recent exposure to As (i.e., within several days) [40]. Data collected from individuals who consume seafood should be interpreted with caution, because their urinary As concentrations may reflect the consumption of organic As compounds, which are less toxic than inorganic As; thus, the As toxicity may be overestimated in seafood consumers [40]. Whole blood or serum is commonly used to assess exposure to Pb and the nutritional status of Se, respectively. Urinary concentrations of Pb and Se are correlated with the corresponding serum/blood concentrations or intake [41–45]; therefore, we treated urinary concentrations of Pb and Se as alternative exposure/intake biomarkers.

The detection limits of urinary metal concentrations (μg/L) were 0.09 for As, 0.08 for Cd, 0.11 for Pb, and 0.44 for Se in this study, corresponding to three times the standard deviation of the blank measurements (*n* = 5). Urinary As and Se were detected in all samples, while the detection rates were 93% and 94% for Cd and Pb, respectively. The Cd and Pb concentrations of the participants in whom these elements were not detected were set at half of the detection limit value.

### Statistical analysis

Of the 1931 participants, we obtained urine samples, anthropometric and blood pressure measurements, and questionnaire data (including sex and age) from 1899 individuals. Their data were used for the statistical analyses (*n* = 1899). There were 541 from Bangladesh, 177 from Indonesia, 690 from Nepal, and 491 from Vietnam.

Creatinine-adjusted urinary concentrations of As, Cd, Pb, and Se were log-transformed to achieve approximately normal distributions; the log-transformed values were used in subsequent analyses. Differences in creatinine-adjusted urinary heavy metal concentrations among the four countries were assessed by one-way analysis of variance, followed by Tukey’s multiple comparison test. Correlations among urinary heavy metal concentrations and those between urinary heavy metal concentrations and blood pressure were investigated by Pearson’s correlation analysis. Following these bivariate analyses, to examine associations between urinary heavy metal concentrations and blood pressure, multiple linear regression models were performed for all participants (covariates: country, age, sex, and body mass index (*BMI*)) and then for the participants in each country. To examine multicollinearity among elements via multiple linear regression analysis, variance inflation factor (VIF) values were calculated; sensitivity analyses were also performed by including interaction terms between correlated elements. Another sensitivity analysis was performed by using the urinary creatinine concentration (log-transformed) as a covariate instead of the creatinine-adjusted urinary heavy metal concentrations, based on a previous suggestion [46]. All analyses were performed using R software (version 4.0.2).

## Results

Table 2 shows the general characteristics of the study participants. There were slightly more female participants (54%) than male participants (46%) in all countries. The medians (interquartile ranges) for age and *BMI* were 37 (29–45) years and 21 (19–23) kg/m^2^, respectively, for all participants. With regard to blood pressure, overall medians (interquartile ranges) were 119 (109–131) and 77 (69–85) mmHg for *SBP* and *DBP*, respectively. Overall, 13% of the participants were considered overweight (25 ≤ *BMI* < 30 kg/m^2^) or obesity (*BMI* ≥ 30 kg/m^2^), while 20% of participants had hypertension (*SBP* ≥ 140 mmHg and/or *DBP* ≥ 90 mmHg).

**Table 2:** General characteristics of all study participants and by country

Characteristics		All countries (*n* = 1899)	Bangladesh (*n* = 541)	Indonesia (*n* = 177)	Nepal (*n* = 690)	Vietnam (*n* = 491)
Sex^a^	Male	874 (46)	258 (48)	90 (51)	300 (43)	386 (48)
	Female	1025 (54)	283 (52)	87 (49)	390 (57)	414 (52)
Age (years)^b^		37 (29–45)	35 (27–45)	41 (35–46)	35 (26–45)	39 (33–46)
*BMI* (kg/m^2^)^b^		21 (19–23)	20 (18–22)	22 (20–24)	22 (19–24)	20 (18–22)
Overweight^a,c^		224 (12)	46 (9)	34 (20)	120 (17)	24 (5)
Obesity^a,d^		27 (1)	3 (1)	5 (3)	17 (2)	2 (0)
*SBP* (mmHg)^b^		119 (109–131)	119 (109–130)	122 (113–136)	118 (108–131)	119 (110–131)
*DBP* (mmHg)^b^		77 (69–85)	75 (68–83)	78 (73–88)	79 (72–88)	75 (68–84)
Hypertension^a,e^		383 (20)	86 (16)	43 (24)	156 (23)	81 (17)

*Abbreviation*s: *BMI*, body mass index; *SBP*, systolic blood pressure; *DBP*, diastolic blood pressure

^a^*n* (%), ^b^median (internal quartile range: quartile 1–quartile 3), ^c^overweight: 25 kg/m^2^ ≤ *BMI* < 30 kg/m^2^, ^d^obesity: *BMI* ≥ 30 kg/m^2^, ^e^hypertension: *SBP* ≥ 140 mmHg and/or *DBP* ≥ 90 mmHg

Table 3 shows the urinary concentrations of As, Cd, Pb, and Se among the participants; creatinine-adjusted values are also shown. In all participants, the geometric means (*GM*s) (geometric standard deviations) of creatinine-adjusted urinary heavy metal concentrations were 84.6 (2.52) μg/g creatinine for As, 0.885 (2.84) μg/g creatinine for Cd, 2.09 (3.63) μg/g creatinine for Pb, and 16.5 (1.64) μg/g creatinine for Se. Participants in Bangladesh had the highest creatinine-adjusted urinary concentrations of As, Cd, and Pb, but had the lowest urinary Se concentrations. The participants in Vietnam had the highest urinary concentrations of Se.

**Table 3:** Urinary metal concentrations of all study participants and by country

Metals	All countries (*n* = 1899)	Bangladesh (*n* = 541)	Indonesia (*n* = 177)	Nepal (*n* = 690)	Vietnam (*n* = 491)
Unadjusted (μg/L)					
Arsenic	56.2 (2.87)	85.0 (2.54)	53.1 (2.16)	26.2 (2.09)	107 (2.64)
Cadmium	0.612 (3.02)	0.786 (2.78)	0.507 (2.56)	0.387 (2.66)	0.942 (3.19)
Lead	1.44 (3.61)	2.80 (2.99)	0.840 (3.72)	1.07 (3.05)	1.29 (4.08)
Selenium	11.0 (2.51)	9.73 (2.13)	18.1 (2.26)	7.23 (2.45)	19.0 (2.23)
Creatinine-adjusted (μg/g creatinine)					
Arsenic^a^	84.6 (2.52)	134 (2.35)^α^	47.7 (1.85)^β^	49.5 (2.01)^β^	133 (2.33)^α^
Cadmium^a^	0.885 (2.84)	1.22 (2.73)^α^	0.436 (2.04)^γ^	0.684 (2.75)^β^	1.15 (2.75)^α^
Lead^a^	2.09 (3.63)	4.41 (3.23)^α^	0.698 (2.89)^δ^	1.95 (2.97)^β^	1.49 (3.67)^γ^
Selenium^a^	16.5 (1.64)	15.3 (1.55)^β^	16.3 (1.37)^β^	13.7 (1.50)^γ^	23.7 (1.71)^α^

Geometric mean (geometric standard deviation)

^a^Results of Tukey’s multiple comparison test. Different letters indicate significant differences for each metal between countries (*α* > *β* > *γ* > *δ*)

Pearson’s correlation coefficients between urinary metal concentrations are shown in Table 4. The strongest correlation was found between As and Se (*r* = 0.39), followed by As and Cd (*r* = 0.34). Other correlations were weaker (*r* = 0.13–0.32), although the coefficients were statistically significant (*p* < 0.001).

**Table 4:** Pearson’s correlation coefficients between the urinary metal concentrations^a^ (*n* = 1899)

	Arsenic	Cadmium	Lead	Selenium
Arsenic	–			
Cadmium	0.31	–		
Lead	0.16	0.32	–	
Selenium	0.39	0.34	0.13	–

^a^Correlation coefficient between log-transformed and creatinine-adjusted concentrations. All the coefficients were statistically significant (*p* < 0.001)

The results of correlation analyses and scatter plots of urinary heavy metal concentrations and blood pressures are shown in Table 5 and Figure S1, respectively. *SBP* was positively correlated with urinary Cd concentration. *DBP* was negatively correlated with urinary As and Se concentrations.

**Table 5:** Correlations between urinary heavy metal concentrations and blood pressure in all participants (*n* = 1899)

	*SBP* (mmHg)		*DBP* (mmHg)	
	*r*^b^	(*95% CI*)	*r*^b^	(*95% CI*)
Arsenic^a^	−0.04	(−0.08, 0.01)	−0.15	(−0.20, −0.11)^***^
Cadmium^a^	0.05	(0.01, 0.10)^*^	0.00	(−0.04, 0.05)
Lead^a^	0.04	(−0.01, 0.08)	0.03	(−0.01, 0.08)
Selenium^a^	−0.02	(−0.06, 0.03)	−0.06	(−0.10, −0.01)^**^

*Abbreviations*: *SBP*, systolic blood pressure; *DBP*, diastolic blood pressure; *CI*, confidence interval

^a^Creatinine-adjusted concentration (μg/g creatinine) (log-transformed)

^b^Pearson’s correlation coefficient

^*^< 0.05, ^**^< 0.01, ^***^< 0.001

Table 6 presents the results of multiple linear regression analyses examining the associations between urinary heavy metal concentrations and blood pressure in all participants (covariates: country, age, sex, and *BMI*). Statistically significant associations were not found between urinary As concentrations and blood pressure. Urinary concentration of Pb was positively associated with both *SBP* and *DBP*, while urinary Se concentration of Se was negatively associated with them. All values of VIF were less than 1.3, so it was unlikely that there was multicollinearity in the multiple linear regression models. Additionally, we performed sensitivity analyses by using urinary creatinine concentration (log-transformed) as a covariate instead of creatinine-adjusted urinary heavy metal concentrations and including interaction terms between either toxic heavy metals (As, Cd, and Pb) or Se; none of the urinary creatinine concentration and the interaction terms was statistically significant, and overall associations between urinary heavy metals and blood pressure did not change (Tables S1 and S2).

**Table 6:** Associations between urinary metal concentrations and blood pressure in all participants (*n* = 1899)

	*SBP* (mmHg)		*DBP* (mmHg)	
	Coef.	(*95% CI*)	Coef.	(*95% CI*)
Arsenic^a^	0.35	(−0.68, 1.38)	−0.27	(−0.96, 0.42)
Cadmium^a^	0.14	(−0.73, 1.02)	0.35	(−0.23, 0.94)
Lead^a^	0.75	(0.07, 1.42)^*^	0.56	(0.11, 1.01)^*^
Selenium^a^	−2.50	(−4.40, −0.61)^**^	−1.60	(−2.88, −0.33)^*^
Age (years)	0.46	(0.39, 0.52)^***^	0.19	(0.15, 0.23)^***^
Sex (ref. = female)	4.64	(3.08, 6.20)^***^	0.60	(−0.44, 1.65)
*BMI* (kg/m^2^)	1.36	(1.12, 1.60)^***^	1.18	(1.02, 1.34)^***^

*Abbreviations*: *SBP*, systolic blood pressure; *DBP*, diastolic blood pressure; *CI*, confidence interval; *BMI*, body mass index

Multiple linear regression models were adjusted by country

^a^Creatinine-adjusted concentration (μg/g creatinine) (log-transformed)

^*^< 0.05, ^**^< 0.01, ^***^< 0.001

**Fig. 1:**
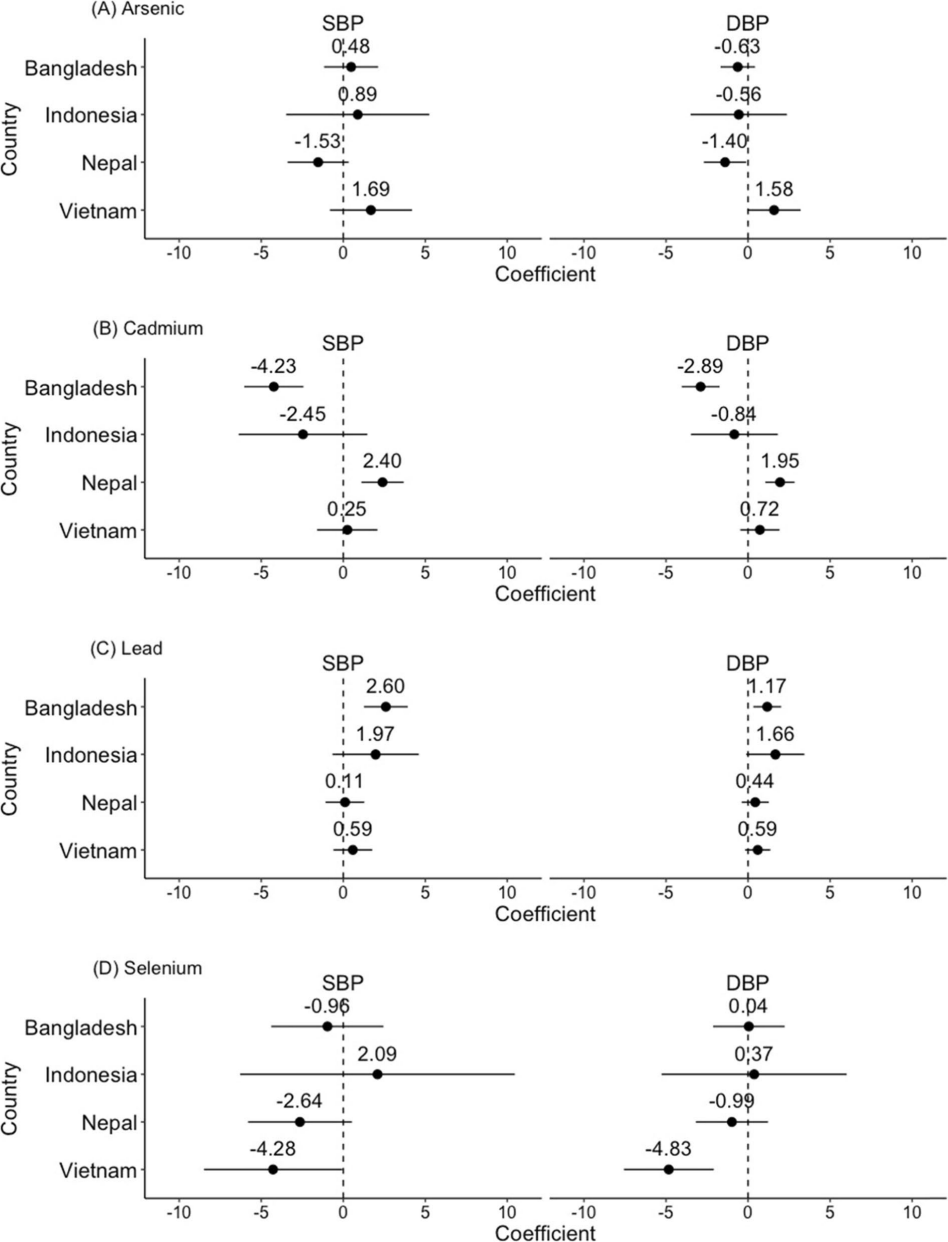
Association between urinary heavy metal concentrations and blood pressure in each country. SBP, systolic blood pressure; DBP, diastolic blood pressure. Bangladesh (*n* = 541); Indonesia (*n* = 177); Nepal (*n* = 690); Vietnam (*n* = 491). Closed circles are coefficients; whiskers represent 95% confidence intervals. All multiple linear regression models included all four heavy metals and were adjusted for age, sex, and body mass index

Figure 1 shows the results of multiple linear regression analyses that evaluated the associations between urinary heavy metal concentrations and blood pressure in each country (see Table S3 for details). Associations between urinary Cd concentrations and blood pressure differed by country: urinary Cd concentrations were positively associated with *SBP* and *DBP* in Nepal, while urinary Cd concentrations were negatively associated with *SBP* and *DBP* in Bangladesh. Other country-specific associations included a negative association between urinary As concentrations and *DBP* in Nepal, a positive association between urinary Pb concentrations and *SBP*/*DBP* in Bangladesh, and a negative association between urinary Se concentrations and both *SBP*/*DBP* in Vietnam.

## Discussion

### Urinary heavy metal concentrations among the participants

Urinary As concentrations observed in the present study (*GM* = 84.6 μg/g creatinine) fell between those reported in As-polluted regions such as in Bangladesh (*GM* = 204 μg/g creatinine for men and 219 μg/g creatinine for women [47]) and China (*GM* = 288.4 μg/g creatinine [48]), and those reported in non-polluted regions such as in the USA (*GM* = 16.9 μg/g creatinine [49]). The urinary Cd and Pb concentrations among participants (*GM*s for Cd and Pb = 0.885 and 2.09 μg/g creatinine, respectively) were comparable to or slightly lower than the values previously reported in non-polluted regions in the east and southeast Asia (*GM*s: Cd = 1.26 μg/g creatinine [50] and Pb = 3.59 μg/g creatinine [51]). We targeted participants who had slightly higher urinary As concentrations and equal or slightly lower urinary Cd/Pb concentrations, compared with those in non-polluted populations. The urinary Se concentrations (*GM* = 16.5 μg/g creatinine) were lower than those reported for regions in Japan (*GM* = 37.5 μg/g creatinine for men and 45.7 for women [52]) or in China (*GM* = 38.3 μg/g creatinine [53]), where Se deficiency is not recognized.

### Associations between urinary heavy metal concentrations and blood pressure

In this study, we examined associations between urinary heavy metal concentrations and blood pressure among residents of four Asian countries. Urinary Pb concentrations were positively associated with both *SBP* and *DBP*, while urinary Se concentrations were negatively associated with them.

Our findings of the positive associations between urinary Pb concentrations and blood pressure agree with previous studies conducted in non-contaminated settings in the USA [54–56], Taiwan [7], and Sweden [22]. Kidney dysfunction due to Pb toxicity is presumably an important contributor to hypertension [10, 57–59]. Increased cardiac output and vasoconstriction are possible effects of Pb exposure [11]. Upon absorption into the human body, Pb can damage endothelial cells by promoting inflammation and/or oxidative stress [11, 12, 60, 61]. Lead can influence the activity of endothelial nitric oxide synthase in blood vessels, thereby suppressing vascular relaxation [11, 62]. Notably, some previous studies have reported no or negative associations between urine or blood Pb concentrations and blood pressure [20, 26]; these inconsistencies could not be explained by differences in the Pb exposure ranges [7, 22, 54–56].

The present study revealed negative associations between urinary Se concentrations and blood pressure. According to a recent systematic review, harmful effects of Se on blood pressure were more likely to occur in populations with high Se intake, while protective effects were observed in Se-deficient populations [13]. Indeed, in a study that involved residents of an area with Se-rich soil and water, positive associations were found between blood pressure and the Se concentrations of serum, nails, and hair [34]. By contrast, in populations likely to include Se-deficient individuals, negative associations between Se intake and blood pressure were found [31, 32, 63]. These negative associations are likely related to the activation of the antioxidative enzyme GPx, for which Se is a cofactor [14, 15]. Increased GPx activity reduces lipid peroxidation, atherosclerotic plaque formation, and platelet aggregation [17–19]. Because the participants in the present study exhibited relatively low urinary Se concentrations, we propose that individuals with higher Se nutritional status could have had increased GPx activity, which potentially contributed to their lower blood pressure.

Two previous studies reported positive associations between urinary As concentrations and blood pressure in As-polluted regions of Bangladesh and China [64, 65]. Other studies also reported positive correlations between urinary As concentrations and blood pressure, including in populations where urinary As concentrations fell within the non-contaminated range [20, 54, 66]. Only one study found a negative association between As concentrations and blood pressure in Argentina [28]. Furthermore, some studies found no association between As concentrations and blood pressure [25, 55], consistent with our results. Although previous studies found that high urinary As concentrations tended to be associated with blood pressure, the direction of the association remains unclear, especially in populations exposed to relatively low levels of As. Inorganic As metabolism varies between individuals because of genetic polymorphisms in the As (+3 oxidation state) methyltransferase gene [67]. This suggests that susceptibility to As toxicity may vary among individuals, which could have led to the conflicting results in the literature.

Unexpectedly, no significant associations were found between urinary Cd concentrations and blood pressure in this study; similar results were reported in a contaminated setting in Bangladesh [25] and in a non-contaminated setting in the USA [54–56], while other studies conducted in non-contaminated settings reported positive [20, 21, 23, 24] and negative [26, 27] associations. The reasons for these inconsistencies among populations have not been clarified.

### Country-specific associations between urinary heavy metal concentrations and blood pressure

A notable finding of this study was that the positive associations between urinary Cd concentrations and *SBP*/*DBP* in Nepal and negative associations in Bangladesh; these differed from the overall trend found in our analyses of all countries. Positive associations between urinary Cd concentrations and blood pressure have frequently been reported in the previous studies [20, 21, 23, 24]; Cd-induced nephrotoxicity [8–10] and/or endothelial damage [12] were proposed as explanations. By contrast, the negative associations, as observed in Bangladesh, have also been found, but in far fewer studies [26, 27]. Although the biological mechanisms contributing to the negative association between urinary Cd concentrations and blood pressure are currently unclear, there are some possible explanations related to kidney function. Urinary Cd concentrations were positively associated with kidney function biomarkers (e.g., estimated glomerular filtration rate) under moderate Cd exposure levels among individuals experiencing high co-exposure to Pb [68]. This implies that urinary Cd excretion is affected by kidney dysfunction due to simultaneous exposure to Pb [69]. As discussed above, kidney dysfunction is a major factor in high blood pressure. Study participants in Bangladesh had higher urinary Cd and Pb concentrations, compared with participants in other countries, and therefore, the negative associations between urinary Cd concentrations and blood pressure could have been caused by confounding kidney dysfunction. Future studies should examine the association between Cd exposure and blood pressure with an additional assessment of kidney status.

In this study, we found several other country-specific associations between urinary As, Pb, and Se concentrations and blood pressure. For example, urinary Pb concentrations were positively associated with *SBP* and *DBP* in Bangladesh, probably because of a higher level of Pb exposure (urinary Pb concentrations were highest in Bangladesh). A systematic review reported that a dose-response relationship between Pb exposure and blood pressure has not been fully characterized [70]. It is possible that Pb affects blood pressure only when the exposure exceeds a certain threshold. The inconsistencies among countries were probably caused by country-specific differences in factors such as nutritional status/intake or physical activity; further investigations are needed to confirm this. In addition, the relatively small sample sizes after stratification by country may have increased the risks of type II errors in the statistical analysis.

### Strengths and limitations

The main strength of this study was that the urine samples were collected in 17 communities with various characteristics across four Asian countries; this enabled an independent investigation of associations between urinary heavy metal concentrations and blood pressure at the local level. Study limitations included measuring the total As concentrations of the urine samples without speciation analysis to distinguish between exposure to toxic inorganic As and non-toxic organic As compounds. In addition, we assessed heavy metal exposure levels using single spot urine samples. To assess the systemic body burdens of Pb and Se, it may have been beneficial to examine their concentrations in whole blood, erythrocytes, or serum/plasma samples [71, 72]. Furthermore, we failed to include some other potential confounders, such as nutrient intakes, smoking/drinking habits, socioeconomic status, and a kidney function marker. Finally, we did not collect information on chronic illnesses, medication, or hospital attendance. The use of anti-hypertensive medications was especially a crucial factor associated with blood pressure, although we speculate that few people used such medications in our study populations based on communications with the village leaders.

## Conclusions

We found that urinary Pb concentration was positively associated with *SBP* and *DBP* in residents of four Asian countries. Notably, this association was present in the context of low urinary Pb concentrations, supporting the toxicity of Pb in terms of blood pressure among populations in non-contaminated areas. In contrast, urinary Se concentrations were negatively associated with *SBP* and *DBP*, implying that Se is protective against increases in blood pressure.

## Supplementary Information

**Additional file 1: Figure S1.** Scatterplot between urinary heavy metal concentrations and blood pressure (n = 1899).

**Additional file 2: Table S1.** Associations between urinary metal concentrations and blood pressure in all participants, with urinary creatinine concentration included as a covariate (n = 1899). **Table S2.** Associations between urinary heavy metal concentrations and blood pressure in all participants: interaction between toxic heavy metals and selenium (n = 1899). **Table S3.** Country-specific associations between urinary heavy metal concentrations and blood pressure.

## Data Availability

The datasets used and/or analyzed during the current study are available from the corresponding author on reasonable request.
