# Supplementary material for: Associations between urinary heavy metal concentrations and blood pressure in residents of Asian countries
Source: Environ Health Prev Med. 2021 Oct 8;26:101. doi: 10.1186/s12199-021-01027-y (PMC8501740; doi:10.1186/s12199-021-01027-y)
Supplement: Supplementary file 1 — Additional file 1: Figure S1. Scatterplot between urinary heavy metal concentrations and blood pressure (n = 1899). [file 12199_2021_1027_MOESM1_ESM.docx]

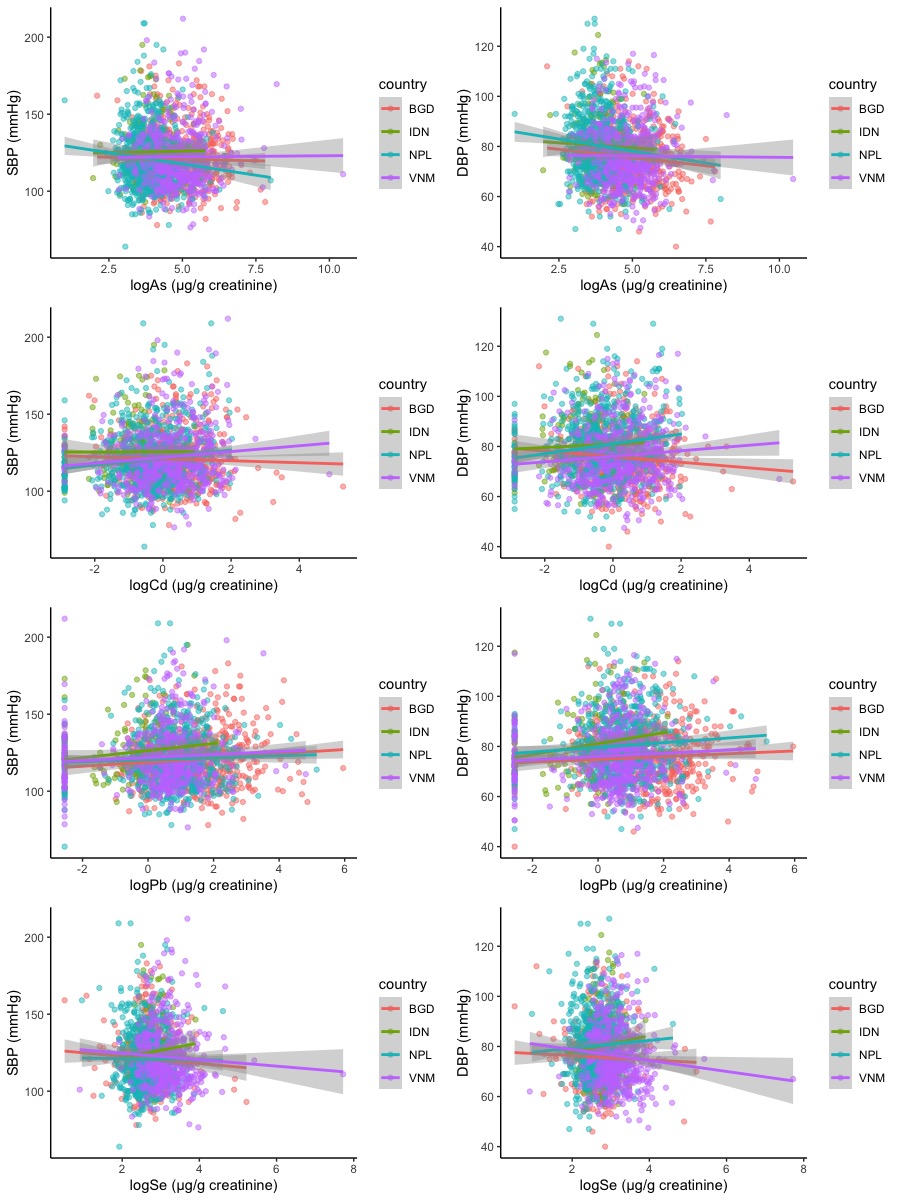


Figure S1 Scatterplot between urinary heavy metal concentrations and blood pressure (n = 1899).

BGD, Bangladesh (n = 541); IDN, Indonesia (n = 177); NPL, Nepal (n = 690); VNM, Vietnam (n = 491); SBP, systolic blood pressure; DBP, diastolic blood pressure. Solid lines present linear regression lines in each country and grey background shows 95% confidence interval of each regression line.
