# Supplementary material for: Associations between urinary heavy metal concentrations and blood pressure in residents of Asian countries
Source: Environ Health Prev Med. 2021 Oct 8;26:101. doi: 10.1186/s12199-021-01027-y (PMC8501740; doi:10.1186/s12199-021-01027-y)
Supplement: Supplementary file 2 — Additional file 2: Table S1. Associations between urinary metal concentrations and blood pressure in all participants, with urinary creatinine concentration included as a covariate (n = 1899). Table S2. Associations between urinary heavy metal concentrations and blood pressure in all participants: interaction between toxic heavy metals and selenium (n = 1899). Table S3. Country-specific associations between urinary heavy metal concentrations and blood pressure. [file 12199_2021_1027_MOESM2_ESM.docx]

| Table S1 Associations between urinary metal concentrations and blood pressure in all participants, with urinary creatinine concentration included as a covariate (n = 1899) | | | | |
| --- | --- | --- | --- | --- |
|  | SBP (mmHg) | | DBP (mmHg) | |
|  | Coef. | (95% CI) | Coef. | (95% CI) |
| Arsenic *^a^* | 0.04 | (-1.05, 1.13) | -0.38 | (-1.11, 0.35) |
| Cadmium *^a^* | -0.13 | (-1.11, 0.84) | 0.20 | (-0.46, 0.85) |
| Lead *^a^* | 0.78 | (0.04, 1.51) ^*^ | 0.60 | (0.11, 1.09) ^*^ |
| Selenium *^a^* | -2.27 | (-4.17, -0.37) ^*^ | -1.47 | (-2.74, -0.20) ^*^ |
| Age (years) | 0.46 | (0.40, 0.53) ^***^ | 0.19 | (0.15, 0.24) ^***^ |
| Sex (ref. = female) | 4.72 | (3.16, 6.29) ^***^ | 0.62 | (-0.43, 1.67) |
| BMI (kg/m^2^) | 1.37 | (1.14, 1.61) ^***^ | 1.19 | (1.03, 1.35) ^***^ |
| Creatinine *^a^* | 0.62 | (-1.31, 2.55) | 0.76 | (-0.53, 2.05) |
| Abbreviation: SBP, systolic blood pressure; DBP, diastolic blood pressure; CI, confidence interval; BMI, body mass index.  Multiple linear regression models were adjusted by country.  *^a^* Log-transformed urinary concentration (µg/L).  ^*^ < 0.05, ^**^ < 0.01, ^***^ < 0.001. | | | | |

| Table S2 Associations between urinary heavy metal concentrations and blood pressure in all participants: interaction between toxic heavy metals and selenium (n = 1899) | | | | |
| --- | --- | --- | --- | --- |
|  | SBP (mmHg) | | DBP (mmHg) | |
| Arsenic and selenium | Coef. | (95% CI) | Coef. | (95% CI) |
| Arsenic *^a^* | 0.36 | (-0.68, 1.39) | -0.25 | (-0.94, 0.44) |
| Cadmium *^a^* | 0.15 | (-0.73, 1.02) | 0.36 | (-0.23, 0.94) |
| Lead *^a^* | 0.75 | (0.07, 1.42) ^*^ | 0.56 | (0.11, 1.01) ^*^ |
| Selenium *^a^* | -2.43 | (-4.40, -0.46) ^*^ | -1.48 | (-2.80, -0.16) ^*^ |
| Age (years) | 0.46 | (0.39, 0.52) ^***^ | 0.19 | (0.15, 0.23) ^***^ |
| Sex (ref. = female) | 4.66 | (3.10, 6.22) ^***^ | 0.63 | (-0.41, 1.68) |
| BMI (kg/m^2^) | 1.36 | (1.12, 1.60) ^***^ | 1.18 | (1.02, 1.34) ^***^ |
| Arsenic × Selenium | -0.12 | (-1.02, 0.77) | -0.21 | (-0.81, 0.38) |
| Cadmium and selenium | Coef. | (95% CI) | Coef. | (95% CI) |
| Arsenic *^a^* | 0.38 | (-0.66, 1.41) | -0.23 | (-0.92, 0.46) |
| Cadmium *^a^* | 0.13 | (-0.75, 1.01) | 0.34 | (-0.25, 0.92) |
| Lead *^a^* | 0.75 | (0.07, 1.42) ^*^ | 0.56 | (0.10, 1.01) ^*^ |
| Selenium *^a^* | -2.42 | (-4.35, -0.49) ^*^ | -1.5 | (-2.79, -0.20) ^*^ |
| Age (years) | 0.46 | (0.39, 0.52) ^***^ | 0.19 | (0.15, 0.23) ^***^ |
| Sex (ref. = female) | 4.66 | (3.10, 6.22) ^***^ | 0.62 | (-0.42, 1.67) |
| BMI (kg/m^2^) | 1.36 | (1.12, 1.59) ^***^ | 1.18 | (1.02, 1.34) ^***^ |
| Cadmium × Selenium | -0.25 | (-1.22, 0.73) | -0.31 | (-0.96, 0.34) |
| Lead and selenium | Coef. | (95% CI) | Coef. | (95% CI) |
| Arsenic *^a^* | 0.36 | (-0.67, 1.40) | -0.26 | (-0.95, 0.44) |
| Cadmium *^a^* | 0.13 | (-0.74, 1.01) | 0.35 | (-0.24, 0.93) |
| Lead *^a^* | 0.76 | (0.09, 1.44) ^*^ | 0.57 | (0.11, 1.02) ^*^ |
| Selenium *^a^* | -2.41 | (-4.33, -0.49) ^*^ | -1.54 | (-2.82, -0.25) ^*^ |
| Age (years) | 0.46 | (0.39, 0.52) ^***^ | 0.19 | (0.15, 0.23) ^***^ |
| Sex (ref. = female) | 4.65 | (3.09, 6.21) ^***^ | 0.61 | (-0.43, 1.65) ^***^ |
| BMI (kg/m^2^) | 1.36 | (1.12, 1.59) ^***^ | 1.18 | (1.02, 1.34) ^***^ |
| Lead × Selenium | -0.28 | (-1.12, 0.56) | -0.20 | (-0.77, 0.36) |
| Abbreviation: SBP, systolic blood pressure; DBP, diastolic blood pressure; CI, confidence interval; BMI, body mass index.  Multiple linear regression models were adjusted by countries.  *^a^* Creatinine-adjusted concentration (µg/g creatinine) (log-transformed and centered).  ^*^ < 0.05, ^**^ < 0.01, ^***^ < 0.001. | | | | |

| Table S3 Country-specific associations between urinary heavy metal concentrations and blood pressure | | | | |
| --- | --- | --- | --- | --- |
|  | SBP (mmHg) | | DBP (mmHg) | |
| Bangladesh (n = 541) | Coef. | (95% CI) | Coef. | (95% CI) |
| Arsenic *^a^* | 0.48 | (-1.17, 2.13) | -0.63 | (-1.68, 0.42) |
| Cadmium *^a^* | -4.23 | (-6.03, -2.42) ^***^ | -2.89 | (-4.05, -1.74) ^***^ |
| Lead *^a^* | 2.60 | (1.27, 3.93) ^***^ | 1.17 | (0.32, 2.01) ^**^ |
| Selenium *^a^* | -0.96 | (-4.38, 2.45) | 0.04 | (-2.13, 2.22) |
| Age (years) | 0.52 | (0.40, 0.64) ^***^ | 0.21 | (0.13, 0.28) ^***^ |
| Sex (ref. = female) | -1.80 | (-4.67, 1.08) | -4.27 | (-6.11, -2.44) ^***^ |
| BMI (kg/m^2^) | 1.02 | (0.56, 1.48) ^***^ | 1.07 | (0.77, 1.36) ^***^ |
| Indonesia (n = 177) | Coef. | (95% CI) | Coef. | (95% CI) |
| Arsenic *^a^* | 0.89 | (-3.48, 5.25) | -0.56 | (-3.50, 2.38) |
| Cadmium *^a^* | -2.45 | (-6.38, 1.48) | -0.84 | (-3.48, 1.81) |
| Lead *^a^* | 1.97 | (-0.66, 4.61) | 1.66 | (-0.11, 3.44) |
| Selenium *^a^* | 2.09 | (-6.29, 10.46) | 0.37 | (-5.28, 6.02) |
| Age (years) | 0.76 | (0.42, 1.09) ^***^ | 0.46 | (0.24, 0.69) ^***^ |
| Sex (ref. = female) | -0.38 | (-6.17, 5.40) | -2.61 | (-6.51, 1.29) |
| BMI (kg/m^2^) | 0.85 | (0.08, 1.63) ^*^ | 0.97 | (0.45, 1.49) ^***^ |
| Nepal (n = 690) | Coef. | (95% CI) | Coef. | (95% CI) |
| Arsenic *^a^* | -1.53 | (-3.39, 0.33) | -1.40 | (-2.70, -0.11) ^*^ |
| Cadmium *^a^* | 2.40 | (1.11, 3.69) ^***^ | 1.95 | (1.05, 2.84) ^***^ |
| Lead *^a^* | 0.11 | (-1.08, 1.29) | 0.44 | (-0.39, 1.26) |
| Selenium *^a^* | -2.64 | (-5.80, 0.53) | -0.99 | (-3.19, 1.22) |
| Age (years) | 0.36 | (0.26, 0.45) ^***^ | 0.13 | (0.06, 0.20) ^***^ |
| Sex (ref. = female) | 8.78 | (6.24, 11.32) ^***^ | 3.27 | (1.51, 5.04) ^***^ |
| BMI (kg/m^2^) | 1.41 | (1.06, 1.77) ^***^ | 1.18 | (0.93, 1.42) ^***^ |
| Vietnam (n = 491) | Coef. | (95% CI) | Coef. | (95% CI) |
| Arsenic *^a^* | 1.69 | (-0.81, 4.19) | 1.58 | (-0.04, 3.21) |
| Cadmium *^a^* | 0.25 | (-1.60, 2.09) | 0.72 | (-0.48, 1.92) |
| Lead *^a^* | 0.59 | (-0.60, 1.77) | 0.59 | (-0.18, 1.36) |
| Selenium *^a^* | -4.28 | (-8.49, -0.06) ^*^ | -4.83 | (-7.57, -2.09) ^***^ |
| Age (years) | 0.62 | (0.47, 0.77) ^***^ | 0.31 | (0.21, 0.41) ^***^ |
| Sex (ref. = female) | 5.53 | (2.45, 8.62) ^***^ | 1.56 | (-0.44, 3.57) |
| BMI (kg/m^2^) | 1.67 | (1.12, 2.22) ^***^ | 1.15 | (0.79, 1.51) ^***^ |
| Abbreviation: SBP, systolic blood pressure; DBP, diastolic blood pressure; CI, confidence interval; BMI, body mass index.  *^a^* Creatinine-adjusted concentration (µg/g creatinine) (log-transformed).  ^*^ < 0.05, ^**^ < 0.01, ^***^ < 0.001. | | | | |
